# Endoplasmic Reticulum Stress-Induced Resistance to Doxorubicin Is Reversed by Mulberry Leaf Polyphenol Extract in Hepatocellular Carcinoma through Inhibition of COX-2

**DOI:** 10.3390/antiox9010026

**Published:** 2019-12-26

**Authors:** Mon-Yuan Yang, Cheng-Hsun Wu, Tung-Wei Hung, Chau-Jong Wang

**Affiliations:** 1 Microbiology and Immunology, Institute of Biochemistry, Chung Shan Medical University, Taichung 402, Taiwan; koiyung@gmail.com; 2 Department of Anatomy, China Medical University, Taichung 404, Taiwan; chsunwu@mail.cmu.edu.tw; 3 Department of Biochemistry, China Medical University, Taichung 404, Taiwan; 4 Department of Medicine, Division of Nephrology, Chung Shan Medical University Hospital, Taichung 402, Taiwan; a6152000@ms34.hinet.net; 5 School of Medicine, Chung Shan Medical University, Taichung 402, Taiwan; 6 Department of Health Diet and Industry Management, Chung Shan Medical University, Taichung 402, Taiwan; 7 Department of Medical Research, Chung Shan Medical University Hospital, Taichung 402, Taiwan

**Keywords:** mulberry leaves, ER stress, drug resistance, hepatocellular carcinoma, mulberry leaf polyphenol extract

## Abstract

Mulberry (*Morus alba* L.) leaves are used in Chinese medicine to treat metabolic disorders. Mulberry leaf polyphenol extracts (MLPE) have recently been shown to exhibit anticancer properties. Endoplasmic reticulum (ER) stress represents a pivotal obstacle in solid tumors, resulting in the antiapoptosis of tumor cells and drug resistance. In this study, pretreatment with the ER stress inducer tunicamycin (TM) attenuated the percentage of apoptosis induced by doxorubicin (DOX). Cotreatment with tunicamycin and MLPE reversed apoptosis induced by DOX. Simultaneously, induction of ER stress with tunicamycin resulted in an increased expression of Cyclooxygenase 2 (COX-2) and Glucose-regulated protein (GRP78) concomitant with the activation of p38 MAPK/PI3K/Akt in HepG2 cells. Furthermore, the suppression of ER stress with celecoxib or p38 MAPK inhibitor successfully recovered DOX-induced apoptosis. Consistent with the inhibition of COX-2 or p38 MAPK, copretreatment with TM and MLPE drastically recovered cytotoxicity and caspase-3 activation in the presence of DOX. These results reveal that MLPE reduces ER stress-induced resistance to DOX in hepatocellular carcinoma (HCC) cells through downregulation of COX-2- or p38 MAPK-mediated PI3K/Akt pathway.

## 1. Introduction

Hepatocellular carcinoma (HCC) is one of the most severe cancers worldwide [1]. Chemotherapy is the most frequently used treatment for HCC because surgical treatment is no longer an option for advanced stages. The chemotherapeutic agent doxorubicin (DOX) is most widely used alone or in combination with other drugs for clinical treatment. However, the development of drug resistance is the major cause of chemotherapy failure in patients with HCC [2]. Therefore, developing strategies to overcome drug resistance in HCC treatment is crucial. 

Endoplasmic reticulum (ER) stress response constitutes a cellular process that can trigger an imbalance in intracellular homeostasis and hamper proper cell functioning [3]. ER stress leads to the activation of an adaptive response, namely, unfolded protein response (UPR), which is aimed at limiting misfolded protein accumulation, enhancing protein clearance, and augmenting ER folding capacity. Prolonged UPR activation initiates cell death mechanisms [4]. However, studies have indicated that ER stress-mediated activation of UPR occurs in cancer tissues [5]. Glucose-regulated protein (GRP78), an ER molecular chaperone, is upregulated when ER stress is induced, and it is also overexpressed in many cancers [6,7]. The effect of GRP78 expression on drug resistance mainly involves reduced drug-induced cell death [8,9]. Tunicamycin is an ER stress inducer, and studies have shown that tunicamycin enhances COX-2 expression in various cell types [10]. Previous studies have indicated that COX-2 is a part of ER stress in many cell types, including chondrocytes [11], head and neck squamous cell carcinoma (HNSCC) [12], and breast cancer [13]. Therefore, the degree of ER stress may play a vital role in the regulation of cell death or survival.

Plant polyphenols are suggested to be associated with a low risk of cancer [14,15]. Mulberry (*Morus alba* L.) leaves are the primary food for silkworms, and they are rich in polyphenols, such as chlorogenic acid, rutin, quercetin, astragalin, and kaempferol, which are considered as strong antioxidants [16]. Mulberry leaf extract has been reported to treat dyslipidemia [17], diabetes [18], and fatty liver [19]. Polyphenols are bioactive molecules that are present in plant-based foods. Dietary intake of plant polyphenols has been associated with several health outcomes related to oxidative stress, including cardiovascular diseases [20], hypertension [21], diabetes [22], mortality [23], and some cancers [24]. The main ingredients of mulberry leaf polyphenol extract (MLPE), such as quercetin, can inhibit the progress of numerous human cancers [25]. Kaempferol is a major flavonoid aglycone that displays several pharmacological properties, including antitumor activity in some types of cancers [26]. The combination of 5-fluorouracil (5-FU) and chlorogenic acid sensitizes hepatocellular carcinoma cells [27]. Anticancer properties of MLPE have been demonstrated in different human cancer cells of the colon [28], breast [28], liver [29], and lung [30]. However, studies investigating the effect of MLPE on ER stress-induced resistance to chemotherapeutic agents in HCC are limited. In this study, we show that MLPE recovers the effects of ER stress-induced resistance to DOX through COX-2- or p38 MAPK-mediated inactivation of the PI3K/Akt pathway.

## 2. Materials and Methods 

### 2.1. Reagents

The COX-2 inhibitor celecoxib, DOX, 3-(4,5-dimethylthiazol-2-yl)-2,5 diphenyltetrazolium bromide (MTT), propidium iodide (PI), and tunicamycin (TM) were obtained from Sigma Chemical (St. Louis, MO, USA). Anti-GRP78 and PI3K antibodies were purchased from BD Biosciences (San Diego, CA, USA). Akt, caspase-3, phosphor-Akt (p-Akt), and phosphor-PI3K (pPI3K) antibodies were obtained from Cell Signaling Technology, Inc (Danvers, MA, USA). Anti-COX-2 antibodies were purchased from Santa Cruz Biotechnology (Santa Cruz, CA, USA). Dulbecco’s modified Eagle medium (DMEM) was obtained from Gibco BRL Life Technologies (Grand Island, NY, USA).

### 2.2. Preparation of MLPE

Mulberry leaves were obtained from Dadu Township, Taiwan. Fresh mulberry leaves were dried immediately and stored at room temperature. For MLPE preparation, 100 g of mulberry leaves in dried powder form was mixed with 300 mL of methanol and heated at 50 °C for 3 h. The extract was filtered and concentrated through evaporation under reduced pressure at room temperature. The extract was resuspended with 500 mL of distilled water and extracted with ethyl acetate. Then, ethyl acetate extract was concentrated through evaporation and lyophilized. MLPE was filtered using a 0.22 μm filter for further use in cell culture.

### 2.3. Cell Culture

A human hepatoma cell line (HepG2, BCRC no. 60025) was purchased from the Bioresource Collection and Research Center (BCRC, Food Industry Research and Development Institute, Hsinchu, Taiwan). Cells were routinely cultured in DMEM supplemented with 10% fetal bovine serum (FBS), 2 mM glutamine, 1.5 g/L of sodium bicarbonate, and 100 U/mL of penicillin-streptomycin. Cells were maintained in a humidified incubator with 5% CO_2_ at 37 °C.

### 2.4. MTT Assay

HepG2 cells were plated into 24-well plates at a density of 5 × 10^4^ cells/well. After 24 h, the culture medium was replaced with various concentrations of MLPE for the indicated time periods. Culture solutions were removed and replaced by a new culture medium. MTT solution (5.0 mg/mL in phosphate-buffered saline (PBS)) was added (20.0 mL/well) to each well, and plates were incubated for 4 h at 37 °C. Subsequently, insoluble formazan crystals were dissolved in 1 mL/well isopropanol and measured spectrophotometrically using a Hitachi U2900 spectrophotometer (Tokyo, Japan) at 563 nm. Viability assays were performed using three independent experiments.

### 2.5. Fluorescence-Activated Cell Sorting 

Cells (1 × 10^6^ cells/mL) were seeded in 6-well plates and then treated with desired concentrations of indicated compounds. After exposure to indicated compounds for specific periods, cells were trypsinized, washed twice with cold PBS, and centrifuged. The pellet was resuspended in 70% ethanol at −20 °C for at least 12 h. Cells were subsequently stained with PI staining and incubated for 30 min in the dark. Cell cycle distribution was analyzed with flow cytometry. The experiment was repeated at least three times.

### 2.6. Western Blotting

After treatment with reagents for 24 h, cells were lysed with RIPA lysis buffer. The lysates were centrifuged, and the supernatant was harvested. Equal amounts of protein samples (50 µg) were subjected to sodium dodecyl sulfate-polyacrylamide gel electrophoresis and electrotransferred onto nitrocellulose membranes (Millipore, Bedford, MA, USA). Membranes were incubated with blocking solution (5% nonfat milk powder with 0.1% Tween 20 in PBS) and then incubated with the indicated primary antibody at 4 °C overnight. Thereafter, membranes were washed three times with 0.1% Tween 20 in PBS and incubated with horseradish peroxidase-conjugated second antibody (GE Healthcare, Little Chalfont, Buckinghamshire, UK). Finally, protein bands were detected using enhanced chemiluminescence and exposed ECL hyperfilm in FUJIFILM LAS-4000 (Tokyo, Japan). Protein quantitation was determined through densitometry using the FUJFILM-Multi Gauge V2.2 software (Tokyo, Japan).

### 2.7. Statistical Analysis

Data represent the mean ± SD of three independent experiments. Statistical analysis was performed using Students’ *t* test. Significance was noted at *p* < 0.05.

## 3. Results

### 3.1. Analysis of MLPE Using High-Performance Liquid Chromatography

MLPE contains abundant polyphenols. According to our previous high-performance liquid chromatography analysis, the constituents of polyphenol in mulberry leaf extract are gallic acid (7.64%), protocatechuic acid (4.69%), catechin (1.2%), gallocatechin gallate (5.88%), caffeic acid (1.02%), epicatechin (0.8%), rutin (1.87%), quercetin (1.24%), and narigenin (2.67%) [31].

### 3.2. Effect of DOX and MLPE on Cell Viability in HepG2 Cells

**Figure 1:**
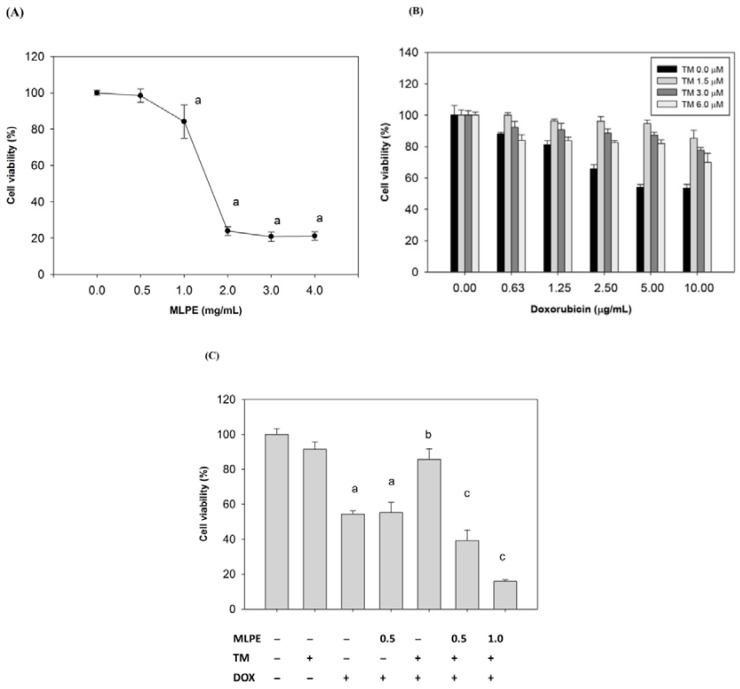

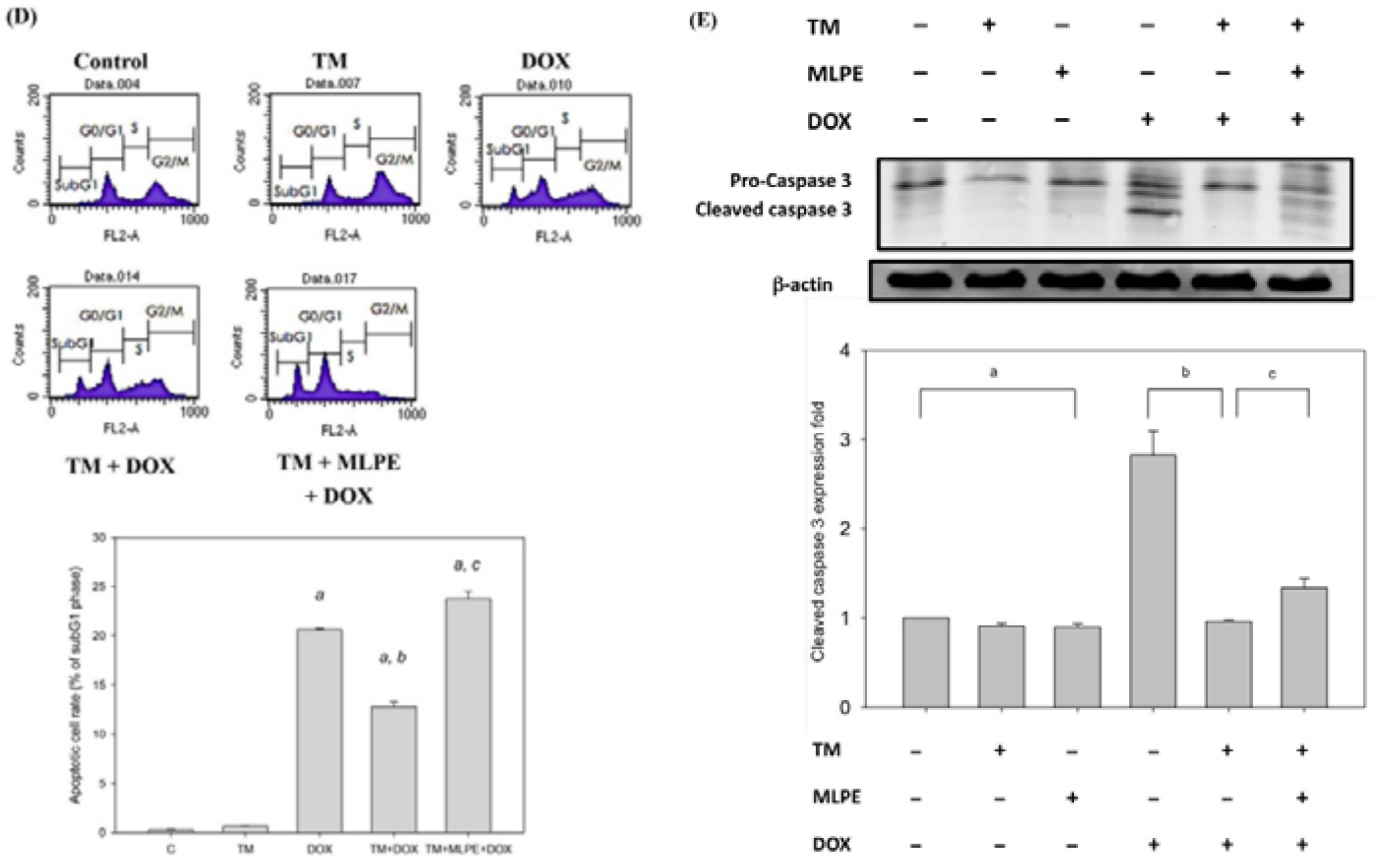
Effect of copretreatment with mulberry leaf polyphenol extract (MLPE) and tunicamycin (TM) on apoptosis induced by doxorubicin (DOX) in HepG2 cells. (**A**) HepG2 cells were pretreated with MLPE (0, 0.5, 1.0, 2.0, 3.0, and 4.0 mg/mL) for 24 h. a, *p* < 0.05, compared with HepG2 cells not treated with MLPE. (**B**) HepG2 cells were treated with TM (0, 1.5, 3, and 6 μM) only for 8 h and then exposed to different concentrations of DOX (0, 0.63, 1.25, 2.5, 5, and 10 μg/mL) for 24 h. Cell viability of HepG2 cells was determined using the 3-(4,5-dimethylthiazol-2-yl)-2,5 diphenyltetrazolium bromide (MTT) assay. Then, HepG2 cells were treated with 1.5 μM TM for 8 h, either in the presence or absence of different concentrations of MLPE, and then exposed to DOX (10 μg/mL) for 24 h. (**C**) Cell viability of HepG2 cells was determined using the MTT assay. (**D**) Apoptosis was analyzed as the subG1 fraction through flow cytometry. The percentage of apoptotic cells in the subG1 phase was examined through flow cytometry. Control, untreated HepG2 cells used as control; TM, HepG2 cells pretreated with tunicamycin; DOX, HepG2 cells treated with DOX alone; TM + DOX, HepG2 cells pretreated with TM and then exposed to doxorubicin; TM + MLPE + DOX, HepG2 cells copretreated with 1.5 μM TM and 0.5 mg/mL MLPE and then exposed to 10 μg/mL DOX. (**E**) Cleaved caspase-3 as an apoptotic marker was measured using Western blotting with a specific anti-caspase-3 antibody. β-actin in the same HepG2 cell extract was used as an internal control. a: *p* < 0.05, compared with HepG2 cells alone; b: *p* < 0.05, compared with HepG2 cells treated with DOX for 24 h; c: *p* < 0.05, compared with HepG2 cells pretreated with TM for 8 h and then exposed to DOX for 24 h. +, means cell treated with the indicated chemical; −, means cell treated without the indicated chemical. Data are expressed as the mean ± SD for three independent experiments.

We initially investigated the cytotoxicity effect of DOX or MLPE on HCC cells (HepG2 cells) using the MTT assay. The viability of HepG2 cells was assessed using various concentrations of MLPE (0.5, 1.0, 2.0, 3.0, and 4.0 mg/mL) and DOX (0, 0.63, 1.25, 2.5, 5.0, and 10.0 µg/mL) for 24 h. As shown in Figure 1A,B, both MLPE and DOX treatment reduced cell viability (1.57 mg/mL and 10 µg/mL IC_50_, respectively) in HepG2 cells. 

### 3.3. Induction of ER Stress Protects HCC Cells against Apoptosis Induced by DOX

To determine the effects of ER stress on DOX-induced cytotoxicity in HepG2 cells, cells were pretreated with TM for 8 h and then subjected to various concentrations of DOX for 24 h. Cell viability was measured by the MTT assay (Figure 1B). Pretreatment with TM significantly reduced DOX-induced cytotoxicity from 0.63 to 10 mg/mL of DOX.

### 3.4. Effect of Copretreatment with MLPE and TM on Apoptosis Induced by DOX in HepG2 Cells

To investigate whether MLPE affects ER stress-induced resistance to DOX, HepG2 cells were treated with 1.5 µM of TM for 8 h in the presence or absence of different concentrations of MLPE (0.5 and 1 mg/mL) and then exposed to DOX (10 µg/mL) for 24 h. Cell viability was determined using the MTT assay. MLPE (0.5 and 1 mg/mL) significantly increased DOX-induced cytotoxicity when HepG2 cells were pretreated with TM (Figure 1C). In addition, MLPE alone without TM pretreatment did not enhance the cytotoxicity of DOX in HepG2 cell (Figure 1C), suggesting that MLPE improved the sensitivity of DOX by inhibiting the effect of ER stress. Cell apoptosis was assessed through flow cytometry and Western blotting. Consistent with the results of the MTT assay, treatment of HepG2 cells with DOX resulted in a marked increase in the sub-G1 phase (20.64%), which was significantly reduced (12.76%) in the presence of tunicamycin. Apoptosis induced by DOX was reversed by copretreatment with TM and MLPE, and the subG1 phase was increased to 22% compared with cells treated with TM (12.76%) (Figure 1D). Apoptosis was also measured through Western blotting of cleaved caspase-3. As shown in Figure 1E, copretreatment with both TM and MLPE significantly increased the levels of cleaved caspase-3 compared with pretreatment with TM alone (Figure 1E). Apoptosis induced by DOX was increased by copretreatment with TM and MLPE. These results support the reversing effect of MLPE on ER stress-induced resistance to DOX.

### 3.5. Protection of HCC Cells against DOX-Induced Apoptosis with Pretreatment of TM is Associated with COX-2

**Figure 2:**
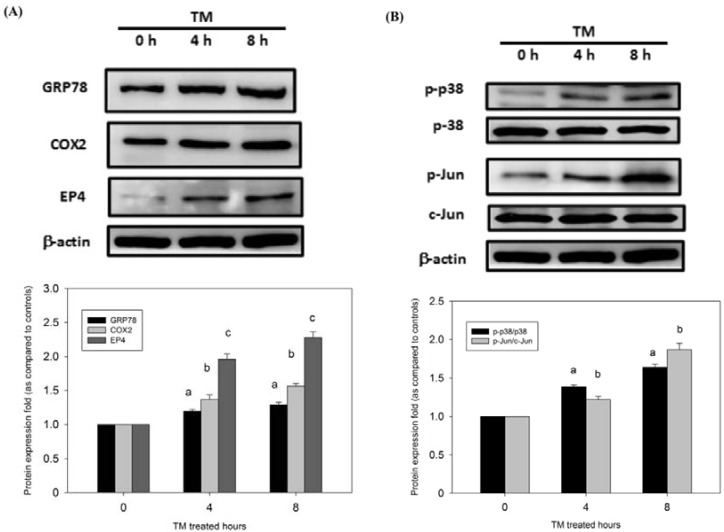

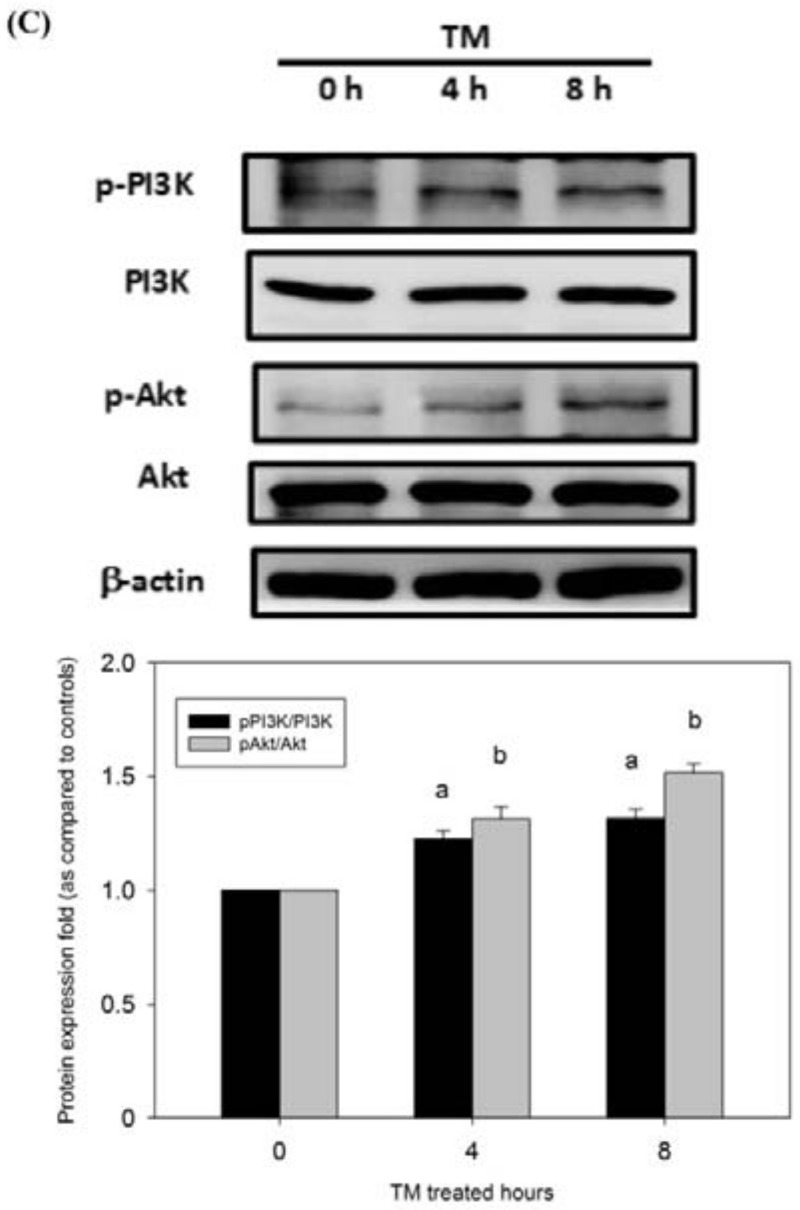
TM treatment induced the endoplasmic reticulum (ER) stress-related protein expression in doxorubicin-induced cells. HepG2 cells were treated with 1.5 μM TM for 0 (control), 4, and 8 h. Equal protein amounts of cell lysates were subjected to Western blotting using a specific antibody as described in the Materials and Methods section. (**A**) COX-2, GRP78 (a hallmark of ER stress), and EP4 were assessed through Western blotting. (**B**) p38/Jun pathway and (**C**) PI3K/Akt pathway were assessed through Western blotting. β-actin in the same HepG2 cell extract was used as an internal control. Optical density reading values of the specific protein versus the loading control protein β-actin are represented as fold of the control values. a, b, c, *p* < 0.05, compared with HepG2 cells alone.

To investigate the mechanisms of TM against DOX-induced apoptosis in HepG2 cells, the protein expression of COX-2, GRP78 (a hallmark of ER stress), and EP4 was assessed through Western blotting. Administration of TM induced an early increase in GRP78 expression, which is indicative of ER stress. The role of ER stress in COX-2 expression in HepG2 cells was investigated. Cells were administrated with TM, which significantly increased the expression of COX-2 and EP4 (Figure 2A). These data indicate that COX-2 is involved in ER stress-induced resistance to DOX.

### 3.6. p38/PI3K/Akt Pathway is Involved in the COX-2-Mediated Cytoprotective Effect of ER Stress against DOX-Induced HCC Cell Apoptosis

To elucidate which signaling pathway is involved in the COX-2-mediated function of ER stress, the phosphorylation of the p38/PI3K/Akt survival pathway was investigated in HepG2 cells. As shown in Figure 2B,C, the expression of phosphor (p)-p38, p-Jun, p-PI3K, and p-Akt rapidly increased after treatment with TM in HepG2 cells. These results show that the p38/PI3K/Akt survival pathway is directly involved in ER stress-induced resistance to DOX.

### 3.7. Effect of Copretreatment with TM and Celecoxib on Cell Survival in HepG2 Cells

**Figure 3:**
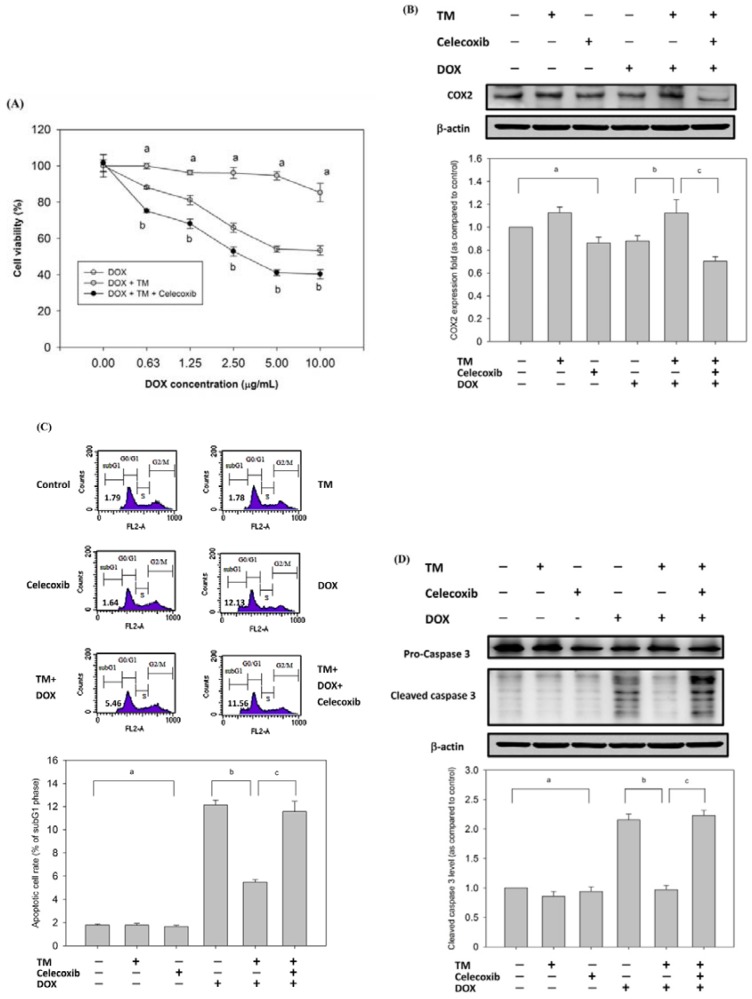

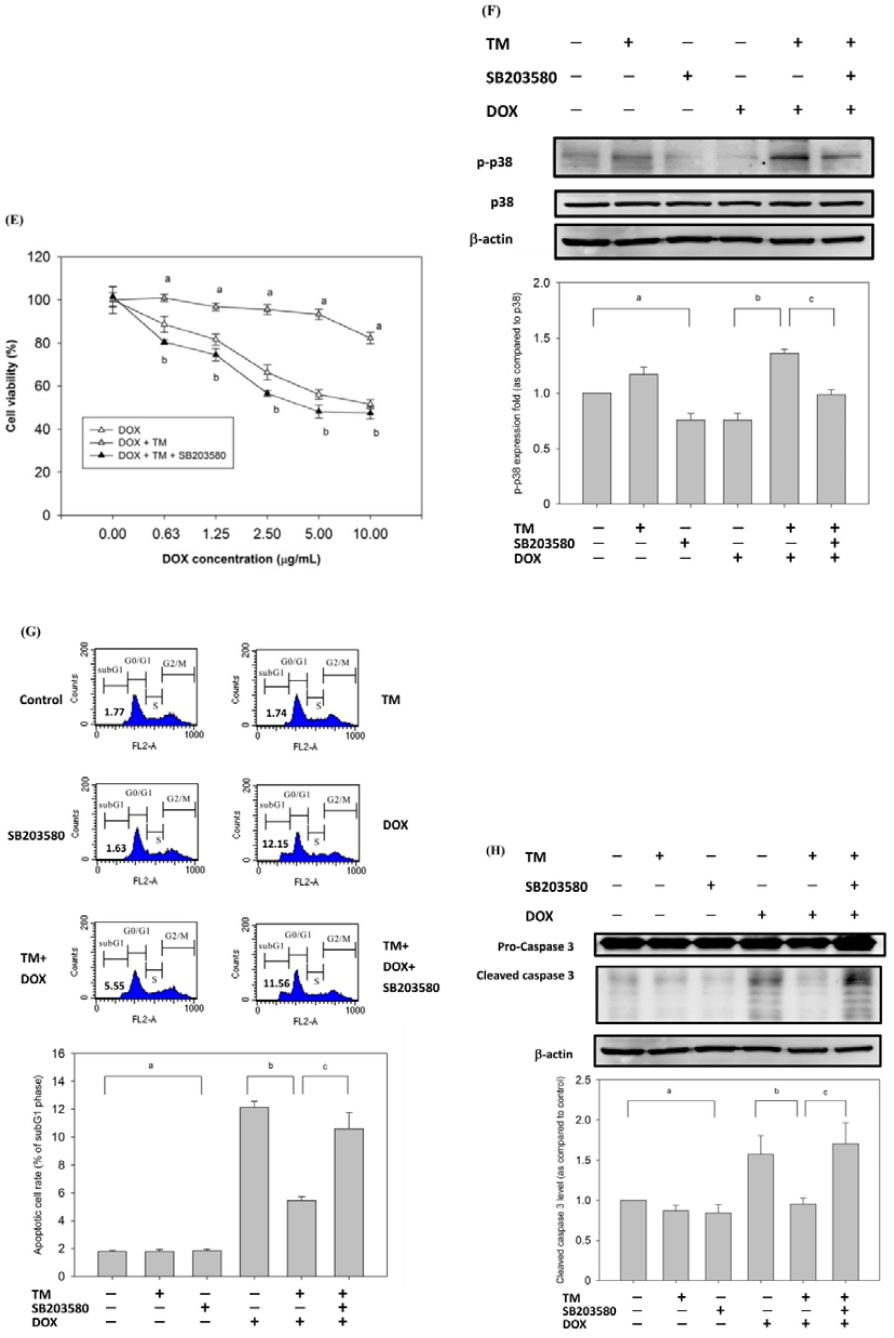
Effect of copretreatment with celecoxib or SB203580 on cell toxicity induced by DOX in tunicamycin-treated HepG2 cells. HepG2 cells were pretreated with 1.5 μM TM for 8 h in the presence or absence of celecoxib (50 μM) and then exposed to DOX (10 μg/mL) for 24 h. (**A**) MTT assay showed the cell viability. Data are expressed as the mean ± SD for three independent experiments. a, *p* < 0.05, compared with HepG2 treated with DOX for 24 h; b, *p* < 0.05, compared with HepG2 pretreated with TM for 8 h and then with DOX for 24 h. (**B**) Cleaved caspase-3 as an apoptotic marker. β-actin was used as an internal control. a, *p* < 0.05, compared with untreated HepG2; b, *p* < 0.05, compared with HepG2 treated with DOX for 24 h; c, *p* < 0.05, compared with HepG2 pretreated with TM for 8 h and then with DOX for 24 h. (**C**) Apoptosis was analyzed as the subG1 fraction through flow cytometry. The percentage of apoptotic cells in the subG1 phase was examined through flow cytometry. Control, untreated HepG2 cells used as control; TM, HepG2 cells pretreated with TM alone; celecoxib, HepG2 cells pretreated with celecoxib alone; DOX, HepG2 cells treated with DOX alone; TM + DOX, HepG2 cells pretreated with TM and then exposed to DOX; TM + celecoxib + DOX, HepG2 cells copretreated with TM and celecoxib and then exposed to DOX. a, *p* < 0.05, compared with HepG2 cells alone; b, *p* < 0.05, compared with HepG2 cells treated with DOX; c, *p* < 0.05, compared with HepG2 cells pretreated with TM and then exposed to DOX. (**D**) Cleaved caspase-3 as an apoptotic marker. β-actin was used as an internal control. a, *p* < 0.05, compared with untreated HepG2; b, *p* < 0.05, compared with HepG2 treated with DOX for 24 h; c, *p* < 0.05, compared with HepG2 pretreatment with TM for 8 h and then treated with DOX for 24 h. HepG2 cells were pretreated with TM for 8 h in the presence or absence of SB203580 (20 μM) and then exposed to DOX for 24 h. Cell viability of HepG2 cells was determined with MTT assay (**E**), Western blotting (**F,H**), and flow cytometry (**G**). Control, untreated HepG2 cells used as control; TM, HepG2 cells pretreated with TM alone; SB203580, HepG2 cells pretreated with SB203580 alone; DOX, HepG2 cells treated with DOX alone; TM + DOX, HepG2 cells pretreated with TM and then exposed to doxorubicin; TM + SB203580 + DOX, HepG2 cells copretreated with TM and celecoxib and then exposed to doxorubicin. a, *p* < 0.05, compared with HepG2 cells alone; b, *p* < 0.05, compared with HepG2 cells treated with DOX; c, *p* < 0.05, compared with HepG2 cells pretreated with TM and then exposed to DOX. +, means cell treated with the indicated chemical; −, means cell treated without the indicated chemical. Data are presented as mean ± SD for three independent experiments.

To confirm that COX-2 enhanced DOX-induced apoptosis, HepG2 cells were pretreated with TM in the presence or absence of celecoxib, a selective COX-2 inhibitor, for 8 h and then exposed to DOX for 24 h. Cell viability was determined using the MTT assay. Celecoxib significantly enhanced cell toxicity when cells were copretreated with TM (Figure 3A). The protein expression of COX-2 was inhibited by celecoxib in HepG2 cells (Figure 3B). Apoptosis was also detected through Western blotting and quantified through flow cytometry. Copretreatment with celecoxib and TM significantly increased the percentage of the subG1 phase and the level of cleaved caspase-3 (Figure 3C,D).

### 3.8. Effect of Copretreatment with TM and SB203580 on Cell Survival in HepG2 Cells

To confirm that p38 is involved in DOX-induced apoptosis, HepG2 cells were pretreated with TM in the presence or absence of SB203580, a selective p38 inhibitor, and then exposed to DOX for 24 h. SB203580 significantly enhanced cell toxicity when copretreated with tunicamycin, as observed through the MTT assay (Figure 3E). The protein expression of p-p38 was inhibited by SB203580 in HepG2 cells (Figure 3F). Flow cytometry showed that copretreatment with SB203580 and TM significantly increased the percentage of the subG1 phase to 10.59% compared to treatment with TM and DOX groups (5.46%) (Figure 3G). Furthermore, the levels of cleaved caspase-3 increased when cells were copretreated with SB203580 and TM groups (Figure 3H). These data suggest that the p38 survival pathway is directly involved in ER stress-induced resistance to DOX.

### 3.9. Reversing Effect of MLPE on ER Stress-Induced Resistance to DOX in HepG2 Cells

**Figure 4:**
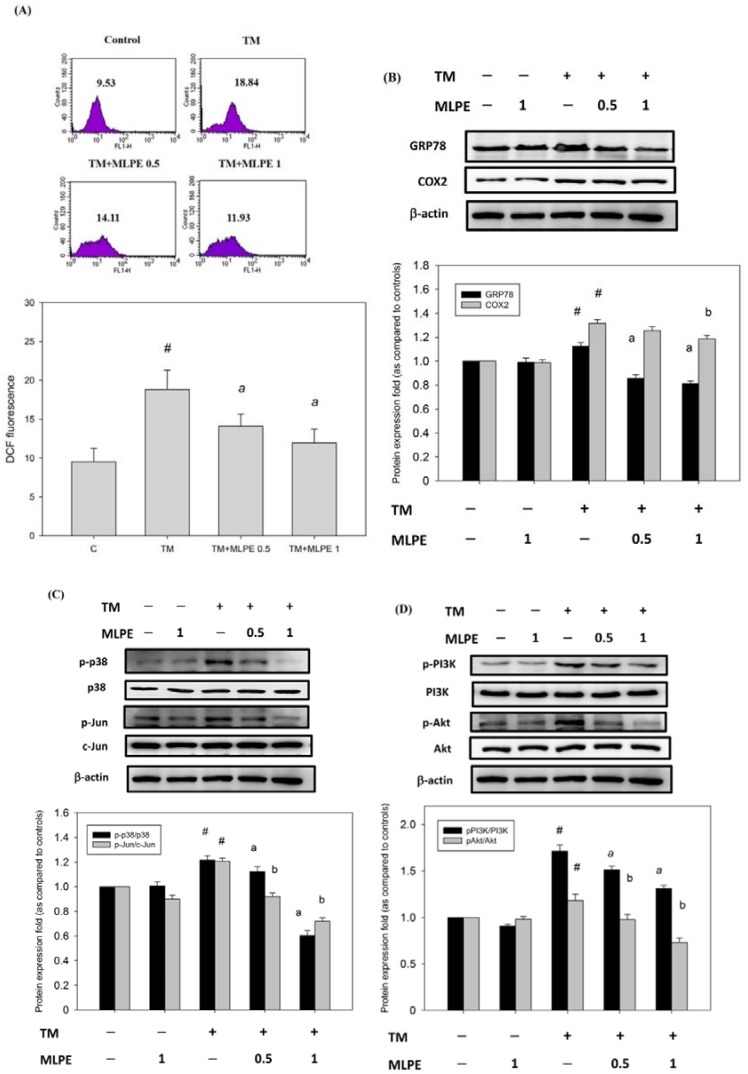
MLPE reduced the level of reactive oxygen species (ROS) and the expression of COX-2, GRP78, p38/Jun, and PI3K/Akt in tunicamycin-induced HepG2 cells. HepG2 cells were treated with TM in the absence (control) or the indicated concentration of MLPE (0.5 and 1 mg/mL) for 8 h. (**A**) Intracellular ROS was detected by dichloro-dihydro-fluorescein diacetate (DCFH-DA) fluorescence. Data were analyzed with flow cytometry. Quantitative assessment was carried out of the DCF fluorescence cell. (**B**) COX-2 and GRP78; (**C**) p38/Jun pathway; (**D**) PI3K/Akt pathway were assessed through Western blotting. Whole cell lysates were subjected to Western blotting analysis. β-actin in the same HepG2 cell extract was used as an internal reference. Optical density reading values of the specific protein versus the loading control protein β-actin are represented as fold of the control values. # *p* < 0.05, compared with untreated HepG2 cells. a, b, *p* < 0.05, compared with HepG2 pretreated with TM for 8 h. +, means cell treated with the indicated chemical; −, means cell treated without the indicated chemical.

A previous study demonstrated that TM treatment increased cellular mRNA and protein expression, which was shown to be mediated by the induction of reactive oxygen species (ROS) [32]. We examined whether the antioxidant effect of MLPE has an inhibitory effect on the ROS level induced by tunicamycin. Results showed MLPE significantly inhibited the levels of ROS induced by TM (Figure 4A). We also determined whether COX-2 or GRP78 levels induced by TM could be altered by MLPE in HepG2 cells. As shown in Figure 4B, MLPE significantly reduced the levels of COX-2 and GRP78 induced by tunicamycin. In addition, the survival signaling pathway, including p-p38, p-Jun, p-PI3K, and p-Akt, was inhibited by MLPE treatment (Figure 4C,D). These data suggest that MLPE exerts an inhibitory effect on ER stress-induced resistance to DOX by targeting the PI3K/Akt pathway via COX-2 or p38.

## 4. Discussion

HCC has been a global health problem, and its incidence is increasing worldwide [33]. Conventional chemotherapy medications, such as DOX, cisplatin, and 5-fluorouracil, have failed to prolong survival because the response rate is very low (from 15% to 20%) [34]. The probability of HCC recurrence is approximately 50% within three years of successful treatment, which has been considered, in part, to be the main mechanism of chemoresistance [35]. This study investigated whether MLPE, a natural herb, can reverse chemotherapy-induced drug resistance in HCC. We first established an ER stress microenvironment, induced resistance to chemotherapy in HepG2 cells, and then exposed the cells to DOX. The results reveal that MLPE can increase the sensitivity of DOX-induced apoptosis in human HCC cells through the COX-2- or p38 mediated PI3K/Akt pathway.

The ER is a compartment of a secretory pathway, such as calcium homeostasis, lipid biosynthesis, and accumulation of folded proteins [3]. Various pathologic stimuli, including hypoxia, alterations in glycosylation status, nutrient deprivation, and oxidative stress, can affect the protein folding process and accumulate unfolded proteins in the ER, activating the UPR [36]. Activation of three UPR sensors, namely, activating transcription factor 6 α (ATF6α), inositol-requiring enzyme 1 α (IRE1α), and protein kinase RNA-activated-like ER kinase (PERK), reduce protein misfolding [37,38,39]. We first mimicked the ER stress microenvironment and investigated DOX-mediated HepG2 cell cytotoxicity and apoptosis. Pretreatment with TM reduced cytotoxicity induced by DOX in HepG2 cells (Figure 1C). Fluorescence-activated cell sorting (FACS) analysis revealed that the SubG1 population increased after DOX treatment but significantly decreased (13.8%) with the presence of TM. Consistent with the FACS data, the cleaved caspase-3 level induced by DOX was decreased in HepG2 cells pretreated with TM (Figure 1E). Our results demonstrated that TM-induced ER stress may cause the resistance of DOX in HepG2 cells.

Severe ER stress induces activation of unique pathways, leading to cell apoptosis. However, studies have indicated that some proteins participate in cell survival in response to ER stress. For example, COX-2 is overexpressed in human breast cancer [13], lung cancer [40], and colon cancer [41] and has been linked to drug resistance [42]. Recent studies have reported that COX-2 is strongly activated in ML-1 and MCF-7 cell lines under ER stress, and the induction is dependent on NF-kB and p38 MAPK [13]. These reports suggest that COX-2 is associated with ER stress induction. Therefore, we investigated the COX-2 status of HepG2 cells during ER stress. We found that exposure to TM increased the expression of COX-2 and GRP78 (Figure 2A). Moreover, when the COX-2 inhibitor celecoxib was used, tunicamycin-mediated protection against DOX was attenuated (Figure 3A–D). Although celecoxib is a selective COX-2 inhibitor, it is the most widely studied agent. Celecoxib has shown potent anticancer activity in animal tumor models [43,44]. Among various COX-2 inhibitors, celecoxib has outstanding anticancer actions. These results indicate that COX-2 sensitizes HCC cells to DOX-induced cell death.

The p38 MAPK signaling pathway regulates multiple cellular processes. p38 MAPK is strongly activated in multiple cells under ER stress [45,46,47]. We examined the p38 activation of HepG2 upon induction of ER stress. Our results showed that p38 MAPK was strongly activated upon ER stress in HepG2 cells (Figure 2B). Apoptosis induced by the combination of SB203580 and TM was higher than that induced by either TM or SB203580 alone (Figure 3G). These data suggest that inhibition of p38 MAPK significantly abrogated the protection of HepG2 cells against DOX-induced apoptosis. The PI3K/Akt signaling pathway has been implicated in the COX-2-mediated cytoprotective function of ER stress [48]. Moreover, we showed that p-Akt/p-PI3K substantially increased upon ER stress induced by TM in HepG2 cells (Figure 2B). 

ER stress is associated with drug resistance. Agents that reverse ER-mediated resistance could define a new strategy for cancer therapy. Mulberry leaf has many types of flavonoids and polyphenolic acid, which can inhibit proliferation and metastasis of multiple tumor cells [28,49]. The effect of MLPE on ER stress-induced resistance to DOX has not been understood. Quercetin, a flavonoid found in MLPE, has been found to enhance apoptosis via ER stress in human cervical carcinoma [50], human ovarian cancer [51], and human colon cancer cells [52]. Rutin, another MLPE flavonoid, has the ability to restore chemosensitivity in human breast cancer cells [53]. Inhibition of ER stress represents a promising therapeutic strategy to attenuate metastasis in different cancer cells. A recent study indicated that mulberry root extract reduces the viability of multi-drug-resistant MCF7/Dox cells [54]. In this study, a combination treatment with MLPE and DOX under ER stress restored DOX-induced apoptosis in tunicamycin-pretreated HepG2 cells. Furthermore, MLPE reduced the expression of COX-2 and subsequently attenuated p38 MAPK/Akt/PI3K phosphorylation in tunicamycin-treated HepG2 cells (Figure 4). These results indicate that the combination of MLPE and DOX causes a strong growth inhibition via the COX2- or p38 MAPK-mediated PI3K/Akt pathway in ER stress-induced resistant HepG2 cells.

## 5. Conclusions

In this study, our findings reveal that MLPE recovers tunicamycin-induced ER stress and then reduces the DOX-mediated apoptosis of HCC cells, indicating that ER stress may be involved in the chemoresistance of doxorubicin. Our results also show that COX2- or p38 MAPK-mediated PI3K/Akt pathway plays an important role in DOX resistance in response to tunicamycin-mediated ER stress. In addition, these findings show that MLPE recovers ER stress-induced resistance to DOX in HCC. In conclusion, the study results suggest that MLPE can be a chemotherapeutic agent in abrogating ER stress-induced resistant HCC cells.
